# Modeling of Hypoxic Brain Injury through 3D Human Neural Organoids

**DOI:** 10.3390/cells10020234

**Published:** 2021-01-25

**Authors:** Min Soo Kim, Da-Hyun Kim, Hyun Kyoung Kang, Myung Geun Kook, Soon Won Choi, Kyung-Sun Kang

**Affiliations:** Adult Stem Cell Research Center and Research Institute for Veterinary Science, College of Veterinary Medicine, Seoul National University, 1 Gwanakro, Gwanak-gu, Seoul 08826, Korea; iamcellist@snu.ac.kr (M.S.K.); dahyun0515@snu.ac.kr (D.-H.K.); gusrudk100@snu.ac.kr (H.K.K.); kmg369@snu.ac.kr (M.G.K.)

**Keywords:** human brain organoid, cerebral cortex, brain ischemia model, neural stem cell, reoxygenation

## Abstract

Brain organoids have emerged as a novel model system for neural development, neurodegenerative diseases, and human-based drug screening. However, the heterogeneous nature and immature neuronal development of brain organoids generated from pluripotent stem cells pose challenges. Moreover, there are no previous reports of a three-dimensional (3D) hypoxic brain injury model generated from neural stem cells. Here, we generated self-organized 3D human neural organoids from adult dermal fibroblast-derived neural stem cells. Radial glial cells in these human neural organoids exhibited characteristics of the human cerebral cortex trend, including an inner (ventricular zone) and an outer layer (early and late cortical plate zones). These data suggest that neural organoids reflect the distinctive radial organization of the human cerebral cortex and allow for the study of neuronal proliferation and maturation. To utilize this 3D model, we subjected our neural organoids to hypoxic injury. We investigated neuronal damage and regeneration after hypoxic injury and reoxygenation. Interestingly, after hypoxic injury, reoxygenation restored neuronal cell proliferation but not neuronal maturation. This study suggests that human neural organoids generated from neural stem cells provide new opportunities for the development of drug screening platforms and personalized modeling of neurodegenerative diseases, including hypoxic brain injury.

## 1. Introduction

Brain ischemia is a serious disease accounting for 5.5 million annual deaths worldwide [1]. This condition leads to insufficient oxygen supply or cerebral hypoxia, inducing the death of brain tissues, cerebral infarction, or ischemic stroke [2]. Ischemic stroke is a cause of long-term disability and is associated with very high rehabilitation costs [3,4]. Because it consumes oxygen at a high rate, the entire central nervous system is very sensitive to changes in oxygen concentration [5]. During hypoxia, the brain resorts to a major adaptive mechanism that allows it to survive. Low oxygen tolerance is thought to stimulate brain plasticity through a combination of energy conservation and improved homeostatic control of subsequent hypoxic damage [6]. During hypoxic injury, modulation works together with plasticity and its supporting function. The mechanisms underlying plasticity and modulation may point to new strategies for preventing and treating hypoxic injuries that may become the focus of clinical studies [7,8,9]. The human body’s ability to adapt to hypoxia has been rigorously investigated [10]. However, the study duration and severity of hypoxia induced in these previous studies were not consistent with what is observed under pathological conditions; therefore, the adaptive response may be different from that reported in these studies. Thus, understanding the cellular mechanism of hypoxic resistance requires more than extrapolation from physiological conditions and can yield unique treatment goals [6]. Recent studies on brain ischemia have paid increasing attention to ethical issues and have used models with minimal involvement of animals [11]. The use of animals models will undoubtedly improve the odds of identifying and developing effective therapeutics [12]. However, the results of these studies cannot determine whether these drugs can treat neurodegenerative diseases in humans or therapeutic studies [13].

In stem cell therapy, neural stem cells (NSCs) have great potential for regenerative treatment of neurodegenerative diseases. Recent studies have shown that mouse and human somatic cells are directly converted to functional and expandable induced NSCs (iNSCs), which have all of the key properties of primary NSCs [14]. Since the directly transformed neuronal population is heterogeneous and cannot proliferate, the induction of NSCs is desirable for obtaining a sufficient number of cells with a relatively safe and homogeneous population of cells. This technique provides an attractive alternative to current-induced pluripotent stem cell (iPSC) technology, as the tumorigenic potential of iNSCs may be significantly lower than that of iPSCs [15]. The direct lineage conversion of differentiated cells into neurons (i.e., induced neurons) or expandable pluripotent iNSCs without passage through the pluripotent phase has been achieved. iNSCs can provide sufficient amounts of neurons and have many uses, such as disease modeling and drug screening [16]. However, there are limitations to stem cell differentiation in monolayer culture. The interaction of the cells with the plastic surface overcomes the interaction between cells or the interaction of cells with the extracellular matrix [17]. The stiffness of plastic plates is not physiologically relevant, and many cells that separate from organs or tumors become flat when they are cultured in monolayers, changing their growth rate and differentiation status. Unsurprisingly, drug screening tests in monolayer cell culture produce different results from those performed in three-dimensional (3D) cell culture [18]. Cells in 3D culture show more complex cell–cell interactions and diversity, reach the later stages of development, and exhibit better function than cells in 2D culture. 3D-cultured cells are called organoids and can be used to more accurately model the cytoarchitecture of organs [19].

Organoids derived from human pluripotent stem cells are widely expected to fill the remaining gaps between animal models and humans, as stem cells derived from humans are the main sources of cultured organoid [20,21]. The development of research platforms using organoid models takes less time than the establishment of animal models. Since human organoids can be generated within weeks or months with a high success rate, organoids derived from patients can be used in the field of personalized medicine to provide compelling personal data, such as information about individualized mutant profiles or drug reactions [22]. While initially identifying incubation conditions for new tissue types is somewhat complex, it is relatively easy for researchers to process large numbers of organoid lines simultaneously [23]. In particular, 3D human brain organoids recapitulate both the anatomy and development of the human cortex [24,25].

Brain organoids generated from pluripotent stem cells offer an alternative research model. For several years, organoid methods have been reported, including the quick reaggregation (SFEBq) method for generating 3D cerebral cortex tissue from pluripotent stem cells using serum-free cultures of floating embryoid body-like aggregates [26]. Subsequently, dissolved Matrigel successfully supports the growth of 3D cortical forebrain tissues from floating SFEBq aggregates [27,28]. 3D brain structure can also be supported by embedding embryoid bodies in pure Matrigel droplets [29,30]. The addition of Matrigel to neural-derived embryonic bodies supports the formation of polarized neuroepithelium with the basal surface facing the environment, such as the external extracellular matrix, and provides the epithelium with support to undergo subsequent morphogenic changes [26]. In vivo, the polarized neuroepithelium of the neural plate folds into itself, forming a round neural tube, a pseudostratified neuroepithelium surrounding a lumen filled with apical fluid [31,32]. The fold of this neural plate has not yet been replicated in organoids, but Matrigel supports the creation of several neural tube-shaped shoots in which neuroepithelial cells are organized in three dimensions around a large apical lumen. When the cortical region of the organoid is compared to a monolayer rosette, the 3D culture system shows a higher level of spatial configuration, exhibiting a ventricular zone, subventricular zone, and intermediate zone, as well as neurons with primitive inside-out layering that develop into deep early-born neurons. The development of this structure is then followed by the generation of later-born neurons that develop layers and ostensibly move into the regions that contain deep early-born neurons [33]. In the body, developing cortical neurons are radially arranged in dense bands called cortical plates. Despite an organoid’s ability to produce basic mobile cortical neurons, adding dissolved Matrigel during the neurogenic stage of organoid development is very important for the production of cortical plates. This may be because the cell substrate of the pial basal membrane, which is produced in a number of nonneural intermediate follicles and does not exist in organoids, is important for proper movement and localization within the nerve plate [34].

In this study, we develop neural organoids from iNSCs because of their self-organization capacity and report a protocol not involving embryoid bodies of different stages or neural induction, which are essential for iPSCs. Instead, we focus on providing conditions for neuroepithelial growth and the environment necessary for neural differentiation and development. These self-organized human neural organoids recapitulate the structure of the human cerebral cortex. Furthermore, we suggest a method for inducing hypoxic brain injury in 3D-cultured neural organoids. In the cortical plate-like domains of hypoxic neural organoids, both cellular components and neuronal maturation were impaired after reoxygenation. These findings were recapitulated in a 3D culture model of hypoxic brain injury and suggest that this human neural organoid platform is valuable for studying proliferation and maturation underlying injury to the developing human cortex.

## 2. Materials and Methods

### 2.1. Culture of Human induced Neural Stem Cells (iNSCs)

The human iNSC lines used in this study were directly reprogrammed from human adult fibroblasts and validated using standardized methods as previously described [15]. In brief, two retroviral factors, SOX2 and HMGA2, were transduced into human dermal fibroblasts. After expansion of these cells, the culture medium was changed to the NSC maintenance medium containing a 1:1 mixture of StemPro NSC SFM (# a1050901, Gibco, Waltham, MA, USA) and ReNcell medium (#SCM005, Sigma-Aldrich, Rowville, Victoria, Australia) comprising 100X GlutaMAX (#35050-061, Gibco), 5 ng/µL FGF-2 (#PHG0261, Gibco), and 5 ng/µL EGF (#PHG0311, Gibco) was used. After neural induction, NSC-like colonies were collected and transferred to a neurosphere culture condition. To establish an iNSC line, cells were cultured as neurospheres and grown as attached cells on PLO/FN-coated dishes, repeatedly. For the passaging, iNSCs were detached with 1 mL StemPro Accutase (#GIB-A11105-01, Gibco) for 3 min, and neurospheres were collected from six-well cell culture plates (#140675, Thermo Fisher Scientific, Waltham, MA, USA). After 5 min, the supernatant was discarded, and 1 mL StemPro Accutase was added. The cells were collected and cultured as free-floating neurospheres in uncoated six-well plates or as monolayers in poly-L-ornithine (#K-P4957-C110, Sigma-Aldrich) and fibronectin (#356008, Corning)-coated six-well plates for ~40 passages.

### 2.2. Generation of Human Neural Organoids from iNSCs

To generate human neural organoids, neurospheres were embedded in 20 µL Matrigel (#354234, Corning, Corning, NY, USA) on day 7. Neurospheres in the droplet were transferred to 60-mm ultra-low attachment culture dishes (#3261, Corning) containing cerebral organoid differentiation medium (CODM). The CODM consisted of a 1:1 mixture of Neurobasal-A (#10888022, Thermo Fisher Scientific) and DMEM/F-12 (#11320082, Gibco), 0.656% insulin (#12585014, Gibco), 1% GlutaMAX (#35050-061, Gibco), 0.5% MEM non-essential amino acids (#11140050, Gibco), 0.0035% 2-mercaptoethanol (#M3148, Sigma-Aldrich), 0.5% N-2 (#GIB-17502-048, Thermo Fisher Scientific), and 1% B-27 minus vitamin A (#GIB-12587-010, Thermo Fisher Scientific). The CODM was replaced every other day. On day 21, polarized neuroepithelium-like structures were transferred to spinner flasks (#3152, Corning) containing CODM comprising 1% B-27 (#GIB-17504-044, Thermo Fisher Scientific). The CODM was replaced every other day for 35 days. To generate mature human neural organoids, the CODM was replaced every other day for 84 days, and the cells were cultured with agitation.

### 2.3. Sample Preparation

Neural organoids were fixed with 4% paraformaldehyde in phosphate-buffered saline (PBS) for 20 min at room temperature. They were washed three times with PBS and then immersed in a 30% sucrose solution overnight. The neural organoids were embedded in 15% sucrose with 7.5% gelatin and slowly frozen in liquid nitrogen in a deep freezer overnight.

### 2.4. Hematoxylin and Eosin Staining

Cryosectioned tissues on silane-coated slides (#5126-20F, Muto, Hongo, Bunkyo-ku, Tokyo, Japan) were washed with running water for 10 min. The slides were stained with hematoxylin (#HX73999849, Merck, Darmstadt, Germany) for 5 min 30 s and then washed with running water for 10 min. Next, the slides were stained with eosin (#3200-2, Muto) for 20 s and dipped three times in distilled water. The stained slides were dipped in 70, 80, 90, and 100% ethanol (#64-17-5, DUKSAN, Ansan-si, Korea) three times each for dehydration. The stained slides were incubated in xylene (#1330-20-7, DUKSAN) for 1 h. The stained slides were sealed with Canada balsam (#8007-47-4, Junsei, Chuo-ku, Tokyo, Japan) and dried overnight at room temperature.

### 2.5. Immunohistochemistry

Slides were washed with 1% acetic acid in PBS comprising 0.025% Triton-X 100 (#X100, Sigma-Aldrich) (PBST) for 20 min. The slides were blocked with blocking solution consisting of 5% normal goat serum in PBST for 1 h. Primary antibodies diluted in blocking solution were applied to the slides overnight at 4 °C. Secondary antibodies were diluted in PBST and applied to the sections for 1 h at room temperature. Alexa Fluor 488 (#A11001, Invitrogen, Waltham, MA, USA)-, 555 (#A21428, Invitrogen)-, and 594 (#A11012, Invitrogen)-conjugated secondary antibodies were used at a 1:500 dilution. DAPI (# D21490, Invitrogen) was diluted 1:1000 in PBST and applied to the slides for 10 min. Finally, the stained slides were mounted with fluorescent mounting medium (#S302380, Dako, Santa Clara, CA, USA) and dried overnight at room temperature. Three images of each slide were captured using a confocal microscope (Eclipse TE200, Nikon, Shinagawa, Tokyo, Japan). The list of antibodies used for immunostaining is provided in Supplementary Table S1.

### 2.6. Analysis of Cell Composition

Neural organoids were immunostained for phospho-vimentin (p-vimentin), SOX2, TBR1, TBR2, and TUJ1. The cortical plates of neural organoids were imaged with a confocal microscope. All TBR1- TBR2-, TUJ1-positive, and double-positive nuclei were individually marked using the “cell counter” plugin in ImageJ. For quantification of organoid cell differentiation, neural organoids were immunostained for SOX2 and p-Vimentin on day 35. Three images of cortical structures from each slide were captured randomly using a confocal microscope. The list of antibodies used for immunostaining is provided in Supplementary Table S1.

### 2.7. Hypoxic Brain Injury Modeling

Neural organoids were exposed to low-oxygen conditions on day 84. The neural organoids were cultured in CODM and transferred to a hypoxic chamber containing 1% oxygen for 48 h. After 48 h, the neural organoids were transferred to a chamber containing 21% oxygen and 5% CO_2_ and cultured in CODM for another 24 h (for reoxygenation).

### 2.8. Statistical Analysis

All values are reported as the mean ± standard deviation (SD) unless otherwise indicated. Statistical analyses were conducted using two-tailed Student’s *t*-test or ANOVA followed by Newman–Keuls post hoc test for multigroup comparisons using GraphPad Prism version 5.0 (GraphPad Software, San Diego, CA, USA). Statistical significance is defined in the figure legends.

## 3. Results

### 3.1. Generation of Three-Dimensional (3D) Human Neural Organoids Derived from iNSCs

**Figure 1:**
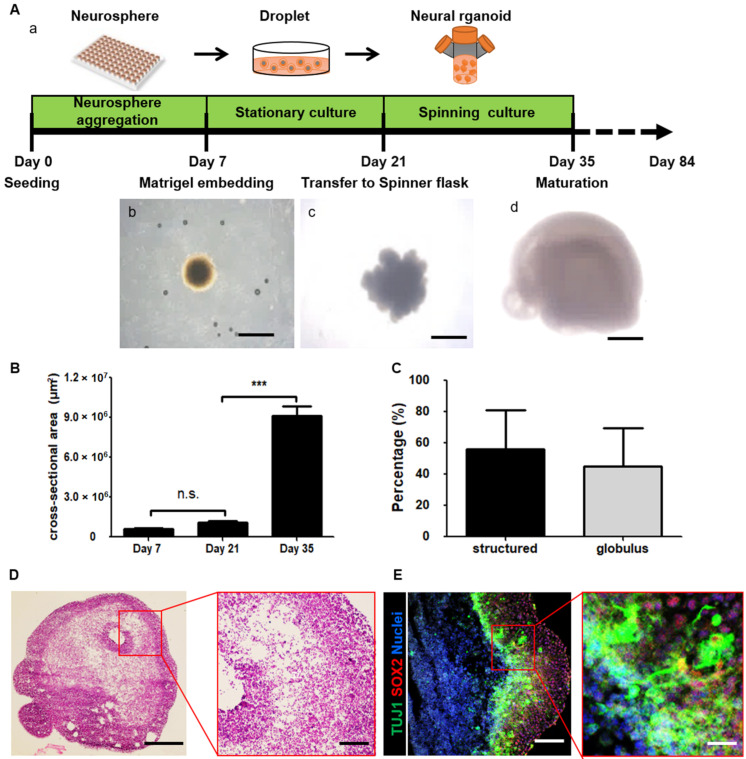
Description of the culture system for three-dimensional (3D) structured human neural organoids derived from induced neural stem cells (iNSCs). (**A**) Schematic overview of the neural organoid generation protocol including a time point of neural organoid culture (a), a neurosphere in a droplet on day 7 (b), an expanded neuroectoderm on day 21 (c), and a neural organoid on day 35 (d). Scale bars, 1 mm. (**B**) Quantification of the cross-section area on days 7, 21, and 35. Each group *n* = 10 organoids, mean ± SD, *** *p* < 0.001, n.s. not statistically significant versus static. (**C**) Quantification of neural organoids on day 35, structured organoid-containing cortex tissue-like structure and folding surface. Globules organoid not folded and contained circle shapes. *n* = 109 organoids, mean ± SD. (**D**) Hematoxylin and eosin staining image of a whole neural organoid on day 35. Scale bars, 1 mm. The inset shows a magnified view in the red square. Scale bars, 200 µm. (**E**) Confocal image shows immature neuron marker TUJ1 (green), radial glia cell marker SOX2 (red), and nuclei (blue) in the neural organoid on day 35. Scale bars, 200 µm. The inset shows a magnified view in the red square. Scale bars, 800 µm.

For 3D culture, we first attempted to generate neurospheres from previously established iNSCs [15]. After seven days, we obtained round neurospheres with a length of 894.1 ± 40.8 µm (Figure 1Ab and Supplementary Figure S1A). As reported in previous studies, the absence of a basement membrane normally leads to a lack of proper orientation and failure of continuous epithelium formation [28]. Therefore, we embedded neurospheres in Matrigel droplets and maintained them in differentiation CODM (Figure 1Aa). On day 21, we observed outgrowth of neuroepithelial buds with a length of 1446.3 ± 153.5 µm outside of the neurospheres (Figure 1Ac and Supplementary Figure S1A). We transferred the neurospheres to a spinner flask to efficiently provide oxygen and nutrients. On day 35, the length of the buds of the neurospheres in spinning flasks increased to 3569.8 ± 815.5 µm (Figure 1Ad and Supplementary Figure S1A).

To further analyze the size of the neurospheres, we measured the cross-sectional area. The cross-sectional areas on days 7, 21, and 35 were 594,962 ± 32,874 µm^2^, 1,062,422 ± 132,914 µm^2^, and 9,077,934 ± 977,968 µm^2^, respectively (Figure 1B). Interestingly, the cross-sectional area significantly increased from day 21 to 35. On days 21 and 35, we identified neurospheres on the basis of morphology (Supplementary Figure S1B,C). On day 35, approximately 60% of neurospheres were identified as 3D neural organoids, as determined by the presence of an expanded neuroepithelium (Figure 1C and Supplementary Figure S1Ca), whereas approximately 40% of neurospheres were not neural organoids, as determined by their globule morphology (Figure 1C and Supplementary Figure S1Cb).

Next, we analyzed the 3D neural organoids. First, the neural organoids were stained with hematoxylin and eosin. The cell density inside the neural organoids was different from that inside the neurospheres (Figure 1D). This finding indicated that iNSCs were stimulated to undergo neural differentiation and subsequent differentiation into specific cell types in the neural organoids. To further investigate the neural tube-like structures in the organoid cross-sections, we stained large continuous cortical tissues within the organoids with the immature neuron marker neuron-specific class III beta-tubulin (TUJ1) and the radial glia cell marker SOX2 (Figure 1E). We found that the 3D neural organoids showed not only an increase in size but also expansion of neuroepithelial morphology. The neural organoids consisted of an organized apical progenitor zone surrounded by basally located neurons.

### 3.2. Neural Organoids Recapitulated the Structure of the Human Cerebral Cortex

In the developing vertebrate brain, elongated bipolar radial glial cells are located at the apical surface of the ventricular zone of the cerebral cortex. These cells migrate through the intermediate zone to the outer cortical plate. Via this inside-out process, early-born neurons occupy the inner layers, while late-born neurons migrate out toward the edge and occupy the superficial cortical layers [35]. In a previous mouse study, the orientation bias of the mitotic spindle was approximately 63% vertical, 33% oblique, and 3% horizontal at E13.5 [36]. In human fetal neocortical tissue, there was an increase in the percentage of horizontally/obliquely oriented mitotic spindles and a decrease in the percentage of vertically oriented mitotic spindles throughout the period of peak neurogenesis in the primate neocortex [37].

**Figure 2:**
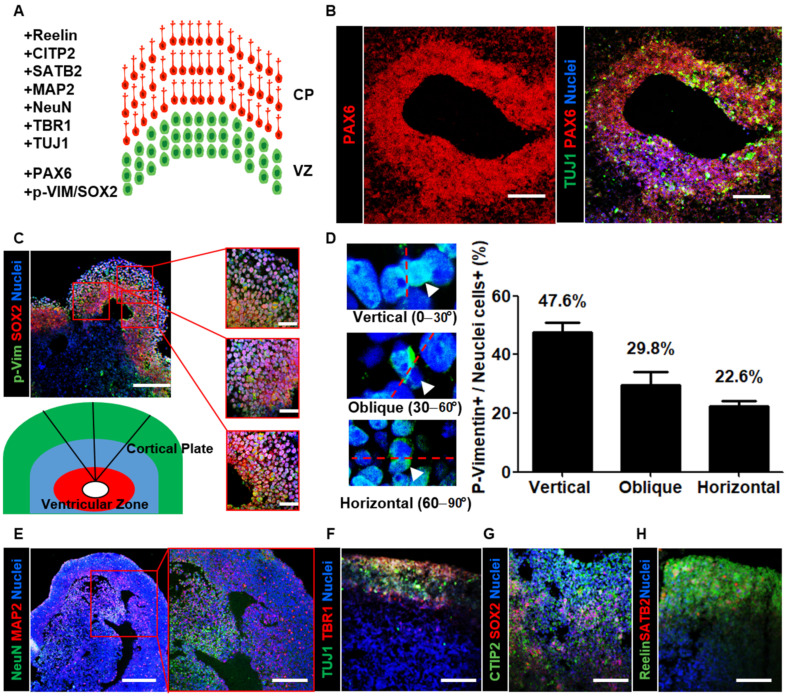
Characterization of neural organoids by layer-specific markers. (**A**) Schematic illustration for the ventricular zone (VZ; green) and cortical plate (CP; red) of neural organoids with layer-specific markers. (**B**) Confocal images show radial glial cells marker PAX6 and immature neuron marker TUJ1 in the ventricular zone-like neural organoid area on day 35. Scale bars, 200 µm. (**C**) Confocal images Scheme 2 and a mitotic marker phospho-Vimentin (p-Vim) in ventricular zone-like and cortical plate-like neural organoid area on day 35. Scale bars, 200 µm. (Right) The insets show a magnified view in three red squares. Scale bars, 100 µm. (Under) Schematic illustration for the neural tube-like zone (red) and cortical plate (green). (**D**) Quantification of vertical (top, left), oblique (middle, left), and horizontal (bottom, left) division planes with dividing p-Vimentin expressing cells in neural organoid. *n* = 84 cells, mean ± SD. (**E**–**H**) Confocal images show cortical layer-specific markers. NeuN (green) and MAP2 (red) (**E**). Scale bars, 250 µm. The inset shows a magnified view in the red square. Scale bars, 200 µm. TUJ1 (green) and TBR1 (red) (**F**), SOX2 (red) and CTIP2 (green) (**G**), SATB2 (red) and reelin (green) (**H**) in neural organoid on day 35. Scale bars, 200 µm.

Here, we analyzed the organization of the cortical region within neural organoids using layer-specific markers (Figure 2A). First, we sectioned neural organoids and found PAX6-enriched apical progenitor zones surrounded by TUJ1-enriched basally located neurons (Figure 2B). To investigate cortical development, we stained organoid cross-sections with both the radial glial cell marker SOX2 and the mitotic marker p-vimentin (Figure 2C). After determining the mitotic spindle orientation, we determined that the percentage of vertically oriented mitotic spindles was 47.6%, which is similar to the orientation bias observed in other mammals (Figure 2D). Furthermore, we identified abundant horizontally/obliquely oriented mitotic spindles (52.4%; obliquely oriented: 29.8%; horizontally oriented: 22.6%). These measurements are consistent with the previously described trend in human fetal neocortical tissue, suggesting that neural organoids recapitulate aspects of cortical development. To further characterize the mature neuronal cells in the outer cortical plate, we stained organoid cross-sections with mature neuron markers. The immunohistochemical data revealed the presence of NeuN- and microtubule-associated protein 2 (MAP2)-positive mature neurons in the outer layer of neural organoids (Figure 2E). Then, we stained the cortical plate with the markers TBR1, CTIP2, SATB2, and Reelin (Figure 2F–H). The data indicated that the localization of these ventricular zone- and cortical plate-specific markers in neural organoids and radial glial cells was similar to that in humans. Furthermore, they suggested that neural organoids recapitulate the structure of the human cerebral cortex.

### 3.3. Neuronal Composition in Developing Neural Organoids

**Figure 3:**
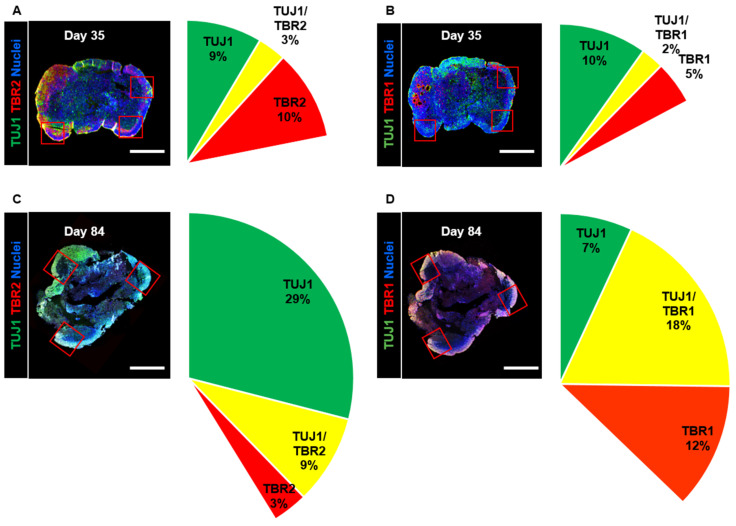
Analysis of the cell composition of developing neural organoids on days 35 and 84. (**A**,**B**) Confocal images showing TUJ1-, TBR2-, and TBR1-positive cell populations in whole neural organoids on day 35. Neurons in the outer layers of neural organoids were counted in three randomly captured confocal images (red squares). Scale bars, 1 mm. The intermediate progenitor marker TBR2 (red), the immature neuron marker TUJ1 (green), the immature neuron marker TBR1 (red), TBR2-/TUJ1-positive cells (yellow), and nuclei (blue) are shown. *n* = 3 in each group. (**C**,**D**) Confocal images showing TUJ1-, TBR2-, and TBR1-positive cell populations in whole neural organoids on day 84. Scale bars, 1 mm. TBR2 (red), TUJ1 (green), TBR1 (red), TBR2-/TUJ1-positive cells (yellow), and nuclei (blue) are shown. *n* = 3 in each group.

As differentiation and development have been proven to take place in the adult human brain [38], we confirmed that these processes occur in long-term-cultured neural organoids. We observed that the size of the organoids did not change between day 35 and day 84 (Supplementary Figure S1A). Then, we stained organoid cross-sections with the intermediate progenitor marker TBR2 and the immature neuron markers TBR1 and TUJ1; we analyzed changes in the cortical projection neuron lineage in neural organoids on days 35 and 84. We measured the expression of the intermediate progenitor marker TBR2 and determined that the percentage of TBR2-positive cells was 10% on day 35, whereas it was 3% on day 84 (Figure 3A,C). Furthermore, the number of TUJ1-positive cells increased from 9% on day 35 to 29% on day 84. These cellular component data indicated a diminished population of intermediate progenitors in the upper layers.

Next, we measured the percentage of cells expressing the immature neuron markers TUJ1 and TBR1, which are specifically expressed in post-mitotic cortical projection neurons. The number of TUJ1-positive cells decreased from 10% on day 35 to 7% on day 84 (Figure 3B,D). However, the number of TBR1-positive cells increased from 5% on day 35 to 12% on day 84. Additionally, the percentage of TBR1- and TUJ1-coexpressing cells was found to be 2% on day 35 but 18% on day 84. The percentage of TBR1-positive cells exhibited a similar increase in the cortical plate. These data indicated that long-term-cultured neural organoids could lead to the development of cortical projection neurons in the cortical plates.

### 3.4. Optimization of a Hypoxic Brain Injury Model in Human 3D Neural Organoids

For the hypoxic brain injury model, we exposed organoids to oxygen and/or glucose deprivation to model ischemia-like conditions in vitro. We first attempted to deprive the organoids of glucose in the culture medium for two days. However, there was no difference in the size or morphology of the neural organoids in the presence and absence of glucose (Supplementary Figure S2A,B). Then, we induced glucose-oxygen deprivation for two days. Unfortunately, glucose-oxygen deprivation led to severe damage, including a decrease in size to 2,427,867 ± 506,927 µm^2^ and loss of layer structures and gene expression of TUJ1 and PAX6 (Supplementary Figure S2A,B). Furthermore, reoxygenation could not reverse these severe effects on neural organoids.

**Figure 4:**
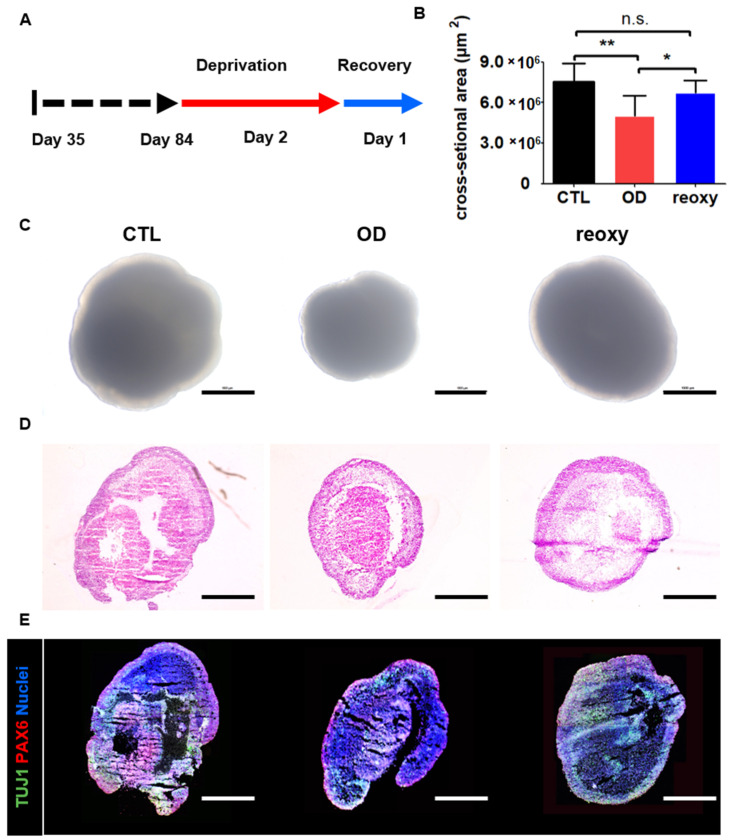
Hypoxic brain injury modeling in human 3D neural organoids on day 84. (**A**) Schematic overview of the hypoxic brain injury protocol, including oxygen deprivation up to day 2 (red arrow) followed by reoxygenation up to day 1 (blue arrow). (**B**) Quantification of the cross-section area (µm2). Control (CTL; black); oxygen deprivation (OD; red); reoxygenation (reoxy; blue). *n* = 5 organoids in each group; mean ± SD; * *p* < 0.05, ** *p* < 0.01, n.s. not statistically significant versus the control. (**C**) Representative phase-contrast images of wild-type organoids (control; CTL), organoids subjected to oxygen deprivation (OD), and organoids subjected to reoxygenation (reoxy). Scale bars, 1 mm. (**D**) Hematoxylin and eosin staining image of CTL, OD, and reoxy organoids. Scale bars, 1 mm. (**E**) Confocal images of staining for an immature neuron marker (TUJ1) and radial glia cell marker (PAX6) in CTL organoids, organoids subjected to OD, and organoids subjected to reoxy. Scale bars, 1 mm.

Next, we studied neural organoids that were cultured under low-oxygen conditions (1%) on day 84 (Figure 4A). After a two-day deprivation period, the size of the neural organoids significantly decreased from 7,455,279 ± 1,363,005 µm^2^ to 5,154,914 ± 1,406,272 µm^2^ under hypoxic conditions (Figure 4B). Moreover, the neural organoids showed a change in shape in phase-contrast images (Figure 4C). This hypoxic damage could also be observed in organoid cross-sections after hematoxylin and eosin staining (Figure 4D). In contrast to glucose-oxygen deprivation, exposure of neural organoids to one day of reoxygenation following exposure to hypoxia restored the organoid size to 6,710,929 ± 1,432,501 µm^2^. However, the layered structures of neural organoids exposed to reoxygenation were not fully restored on day 86, as determined by hematoxylin and eosin staining (Figure 4D). Moreover, immunostaining for the radial glial cell marker PAX6 and immature neuron marker TUJ1 showed diminished staining in inner structures as well as a diminished number of stained neuronal cells in neural organoids exposed to reoxygenation. These staining data indicated a lack of ventricular zone and cortical plate layers in neural organoids after reoxygenation.

### 3.5. Impaired Cellular Components in the Cortical Plate-Like Domains of Hypoxic Neural Organoids after Reoxygenation

**Figure 5:**
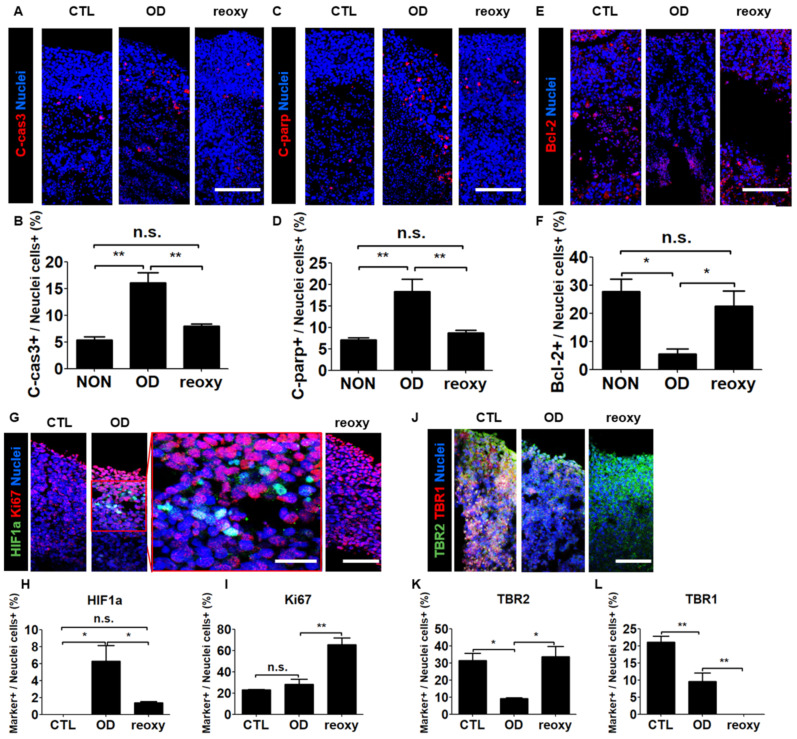
The hypoxic brain injury model recapitulated brain injury and disruption of neuronal cell development after reoxygenation. (**A**–**D**) Tiled confocal images of staining for apoptotic markers C-cas3 (**A**) and C-parp (**C**) in CTL organoids, organoids subjected to OD, and organoids subjected to reoxy. Scale bars, 200 µm. Quantification of C-cas3 (**B**) and C-parp (**D**)-positive cell populations in CTL organoids, organoids subjected to OD, and organoids subjected to reoxy. *n* = 3 in each group; mean ± SD; ** *p* < 0.01, n.s. not statistically significant versus the control. (**E**,**F**) Tiled confocal images of staining for the anti-apoptotic marker bcl-2 (**E**) in CTL organoids, organoids subjected to OD, and organoids subjected to reoxy. Scale bars, 200 µm. Quantification of markers bcl-2 (**F**)-positive cell populations in CTL organoids, organoids subjected to OD, and organoids subjected to reoxy. *n* = 3 in each group; mean ± SD; * *p* < 0.05, n.s. not statistically significant versus the control. (**G**) Tiled confocal images showing staining for a key oxygen-labile protein in the hypoxia pathway, HIF1a, and the proliferation marker Ki67 in wild-type organoids (control; CTL), organoids subjected to oxygen deprivation (OD), and organoids subjected to reoxygenation (reoxy). Scale bars, 200 µm. A magnified view is shown in the red square. Scale bars, 100 µm. (**H**,**I**) Quantification of HIF1a- (**H**) and Ki67-positive (**I**) cell populations in CTL organoids, organoids subjected to OD, and organoids subjected to reoxy. *n* = 3 in each group; mean ± SD; * *p* < 0.05, ** *p* < 0.01, n.s. not statistically significant versus the control. (**J**) Tiled confocal images of staining for the intermediate progenitor marker TBR2 and immature neuron marker TBR1 in CTL organoids, organoids subjected to OD, and organoids subjected to reoxy. Scale bars, 200 µm. (**K**,**L**) Quantification of TBR2 (**K**)- and TBR1 (**L**)-positive cell populations in CTL organoids, organoids subjected to OD, and organoids subjected to reoxy. *n* = 3 in each group; mean ± SD; * *p* < 0.05, ** *p* < 0.01, n.s. not statistically significant versus the control.

To determine the effect of oxygen in the hypoxic human brain injury model, we first performed cell death analysis using by immunostaining with apoptotic markers. Both pro-apoptotic markers c-Cas3 and c-PARP were expressed at the level of approximately 5% in a normal condition, whereas their expression level in a condition of oxygen deprivation was elevated to 16.1% and 18.4%, respectively (Figure 5A–D). In contrast, the anti-apoptotic marker Bcl-2 showed a significant increase after oxygen deprivation and a significant increase following a reoxygenation (Figure 5E,F). Second, we investigated the nuclear localization of hypoxia-inducible factor-1 alpha (HIF1a), a key oxygen-labile protein in the hypoxia pathway. Immunohistochemical analysis revealed the percentage of HIF1a-expressing cells significantly increased to 6.3% under hypoxic conditions and significantly decreased to 1.4% after reoxygenation (Figure 5G,H). In contrast, the percentage of Ki67-positive cells (28.3%) did not change significantly under hypoxic conditions but significantly increased to 65.5% after reoxygenation (Figure 5I). These data showed that reoxygenation significantly increased cell proliferation in neural organoids damaged by exposure to hypoxia.

Next, we investigated the transcriptional changes associated with exposure to hypoxia by performing Real-time-PCR. After oxygen deprivation, the radial glia cell marker *SOX2* and the development neuron marker *SATB2* were significantly downregulated, whereas the expression level of *EMX1* gene was not changed (Supplementary Figure S2C–E). Interestingly, we could observe that the expression level of such genes was significantly increased immediately by reoxygenation. The dorsal forebrain progenitor marker *EMX1* was increased approximately 2.5-fold compared to a Control (Supplementary Figure S2E). As an overlap of the hypoxia-induced differential expression of brain-related regulatory genes in neural organoids, we investigated neuronal maturation in the cortical plate-like domains by immunocytochemistry in organoid cross-sections with the intermediate progenitor TBR2 and the projection neuron marker TBR1 (Figure 5J). Unlike the percentage of HIF1a-positive cells, the percentage of TBR2-positive cells significantly decreased from 31.4% to 9.2% under hypoxic conditions but significantly increased to 33.8% after reoxygenation (Figure 5K). However, the percentage of TBR1-positive cells significantly decreased from 21.1% to 9.6% under hypoxic conditions and significantly decreased from 9.6% to 0% after reoxygenation (Figure 5L). These data indicated that after hypoxic brain injury, reoxygenation could induce neuronal cell proliferation but not neuronal maturation.

## 4. Discussion

Analysis of organoid formation can provide important information concerning human development and organ regeneration and can highlight the value of basic biological research in addition to the potential applications of phosphorus in pharmaceutical drug experiments and molecular medicine. The potential of organoids to better complement existing model systems than basic biological research, medical research, and drug discovery studies, and to be investigated in environments that are physiologically relevant to humans is increasingly being recognized [23].

As is well-known, ischemic stroke occurs when an artery that supplies blood to the brain is blocked. In infants, Hypoxic-ischemic encephalopathy, also known as birth asphyxia, can arise from oxygen deprivation, which leads to prematurity of the human brain. Conventional modeling of hypoxia is limited to cell differentiations on plastic surface in a monolayer culture. Recently, the technical difficulties were overcome, and the 3D organoid cultures emerged as a novel model system. This 3D cell culture technique enables to resemble the developing human brain including the interaction of cells with the extracellular matrix. In the low oxygen condition, human neural organoids revealed the expected nuclear localization of the HIF-1a protein. As expected, this hypoxia-inducible factor was destabilized by reoxygenation.

In this study, we generated self-organized human neural organoids from adult dermal fibroblast-derived iNSCs. These 3D human neural organoids recapitulated developing human cortical plate-like domains. Remarkably, we generated 3D brain organoids from human NSCs cultured in vitro in an attached monolayer under spinning conditions, which is very useful for neurodegenerative disease modeling. Based on our results, we suggest that human neural organoids could mimic the features of the human cerebral cortex and that oxygen deprivation could induce hypoxic brain injury accompanied by disruption of neuronal cell components. Furthermore, it seems that reoxygenation leads to increased neuronal cell proliferation but cannot restore neuronal maturation in human neural organoids. However, glucose-oxygen deprivation led to severe damage, so that reoxygenation could not reverse these severe effects on neural organoids. The glucose deprivation could not lead to a significant decrease in size of neural organoids, whereas the low oxygen led to a significant decrease in size of neural organoids to 5,154,914 ± 1,406,272 μm^2^ (Supplementary Figure S2B). Remarkably, the glucose-oxygen deprivation could lead to a dramatic decrease in size of neural organoids to 2,427,867 ± 506,927 μm^2^. Therefore, the reoxygenation could not restore the decreased size to a normal range of 7,455,279 ± 1,363,005 μm^2^. In addition, these phenomena have been observed in loss of layer structures and gene expression of TUJ1 and PAX6 in organoid cross-sections.

In terms of stem cell biology, TUJ1, TBR2, and TBR1 are expressed sequentially in the cortical projection neuron lineage [39]. TUJ1-enriched neurons indicate the initiation of cortical development from neural stem cells, whereas intermediate progenitors express TBR2 and then TBR1. An analysis of maker distribution across the ventricular zone / sub ventricular zone/cortical plate can be considered cortex-specific ‘transit-amplifying cells’ with a finite life span that arises from proliferating and differentiating stem cells [40]. The subsequent transition from intermediate progenitor cells to projection neurons is characterized by the downregulation of TBR2 and an upregulation of TBR1, which are specifically expressed in post-mitotic cortical projection neurons.

Nevertheless, we need to generate more complex human cortex organoids with a central nervous system and blood vessels. In a recent study, cerebral organoids were implanted into nonobese diabetic/severe combined immunodeficient mice for vascularization [41]. The researchers developed an efficient in vivo engraftment model of human pluripotent stem cell-derived brain organoids in a physiological tissue environment. The transplanted organoids were easily integrated into the mouse brain, showed a gradual temporal differentiation pattern of nerve cells, developed a functional vasculature system, and produced mature and functional human brain tissue in vivo that responded to physiological stimuli triggered by anesthesia. The organoids showed unprecedented axonal growth. The researchers used photogenetics to demonstrate functional synaptic connectivity between the transplanted organoids and the host brain [41,42]. Another recent study used *ETV2* gene-overexpressing iPSCs to generate brain organoids with blood vessels in a circular culture system [43]. Reprogramming *ETV2*-induced endothelial cells (ECs) from organoids is an efficient way to generate vascularized human cortical organoids (vhCOs). Networks similar to functional vascular structures allow us to investigate brain development and disease mechanisms, making them very useful platforms [44,45,46]. This vhCO model system shows that neural and EC interactions are capable of more physiological expression than the brain, reducing the cellular arthrosis and hypoxic state of internal tissues containing typical blood-free organoids [43].

In addition, our neural organoid system does not consist of immune cells. The inflammatory component of encephalopathy in humans is not captured here [47]. Organoid models could incorporate microglia and other cells to study their contributions to human encephalopathy [48]. hCS derivation and maintenance are pursued at atmospheric oxygen levels. Our model does not include cortical circuits [49]. Therefore, future studies could use assembly organoid models that combine dorsal and ventral forebrain organoids to model migration [50]. Similarly, forebrain organoids, including oligodendrocytes, astrocytes, and neurons, can be subjected to the hypoxia model we describe to assess myelination defects [51].

In conclusion, our study indicated that this method can be used to model human neural development and neurodegenerative diseases, and provides a new platform for screening drugs for clinical trials for brain ischemia. This model will be useful for studying a variety of neurodegenerative diseases and neural development in the human brain. We exposed neural organoids to low-oxygen concentrations in vitro and found a decrease in the number of a specific group of cortical progenitors that are thought to contribute to expanding the human cerebral cortex. This model recapitulates not only human cortical development and differentiation of NSCs but also the development of the human brain after hypoxic injury.

## Data Availability

There are no data relevant to accession codes or unique identifiers that are not publicly available. All generated data are included in the manuscript and available by reasonable request to either S.W.C. or K.-S.K.

## Supplementary Materials

The following are available online at https://www.mdpi.com/2073-4409/10/2/234/s1, Figure S1: Neurosphere, neuroectoderm, and 3D structured neural organoid length and morphology, Figure S2: Optimization of a brain ischemia model using human 3D neural organoids, Table S1: List of Antibodies.

## Abbreviations

CODM	cerebral organoid differentiation medium
CP	cortical plate
CTL	control
ECs	endothelial cells
hCS	human Cortical Spheroids
i.e.,	id est; That is
iNSCs	induced neural stem cells
iPSCs	induced pluripotent stem cells
NSCs	neural stem cells
OD	oxygen deprivation
OGD	oxygen glucose deprivation
PBS	phosphate buffered saline
PBST	PBS with 0.025% Triton-X100
p-vimentin	phospho-vimentin
reoxy	reoxygenation
n.s.	not statistically significant versus Static
MAP2	microtubule-associated protein 2
SD	standard deviation
SFEBq	serum-free floating culture of embryoid body-like aggregates with quick reaggregation
SVZ	sub ventricular zone
3D	three-dimensional
TUJ1	neuron-specificclass III beta-tubulin
VZ	ventricular zone
vhCOs	vascularized Human cortical organoids
