# Supplementary material for: Modeling of Hypoxic Brain Injury through 3D Human Neural Organoids
Source: Cells. 2021 Jan 25;10(2):234. doi: 10.3390/cells10020234 (PMC7911731; doi:10.3390/cells10020234)
Supplement: Supplementary file 1 [file cells-10-00234-s001.pdf]

Supporting Information

Modeling of Hypoxic Brain Injury Through 3D Human Neural Organoids

Min Soo Kim[1], Da-Hyun Kim[1], Hyun Kyoung Kang[1], Myung Geun Kook[1], Soon Won Choi[1,*] and Kyung-Sun Kang[1,*]

Adult Stem Cell Research Center and Research Institute for Veterinary Science, College of Veterinary Medicine, Seoul National University, 1 Gwanakro, Gwanak-gu, Seoul 08826, Republic of Korea

Supplementary Materials

Figure S1. Neurosphere, neuroectoderm, and 3D structured neural organoid length and morphology.

Figure S2. Optimization of a brain hypoxic model using human 3D neural organoids.

Table S1. List of Antibodies.

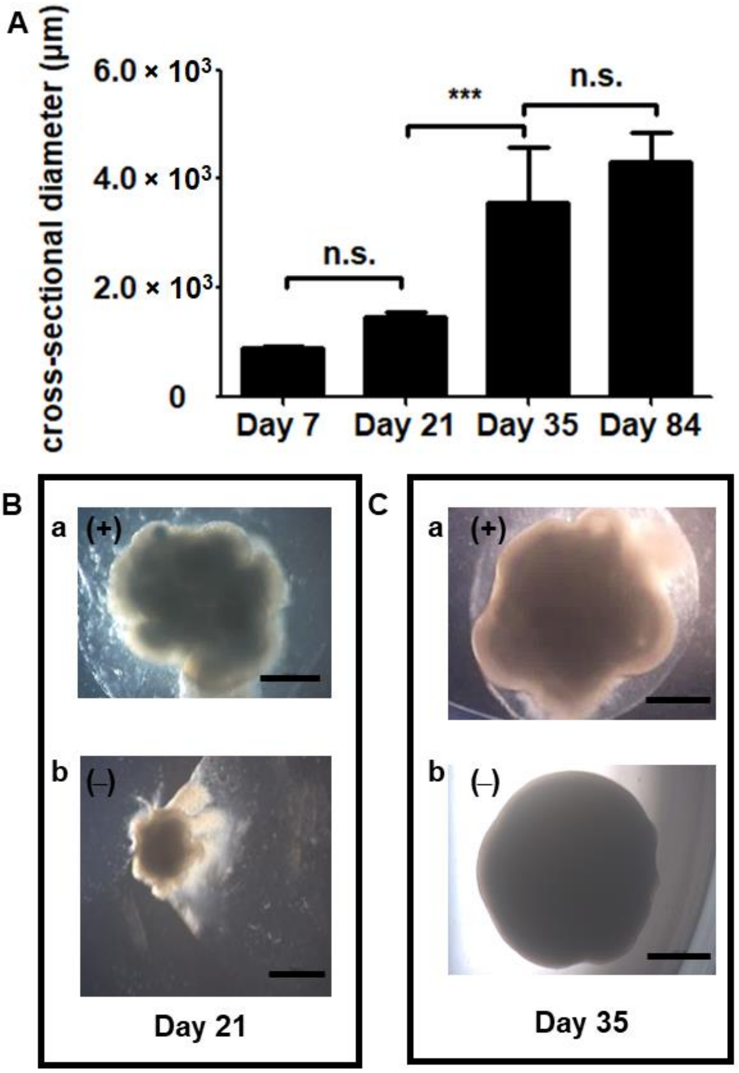

Supplementary Figure S1. Neurosphere, neuroectoderm, and 3D neural organoid length and morphology (**A**) Quantification of the cross-section diameter (μm) on days 7, 21, 35, and 84. n = 10 organoids in each group; mean ± SD; ***p < 0.001, n. s. not statistically significant versus the control. (**B–C**) Representative phase-contrast image of positive (Ba,Ca) and negative (Bb,Cb) organoids on days 21 and 35. Scale bars, 1 mm.

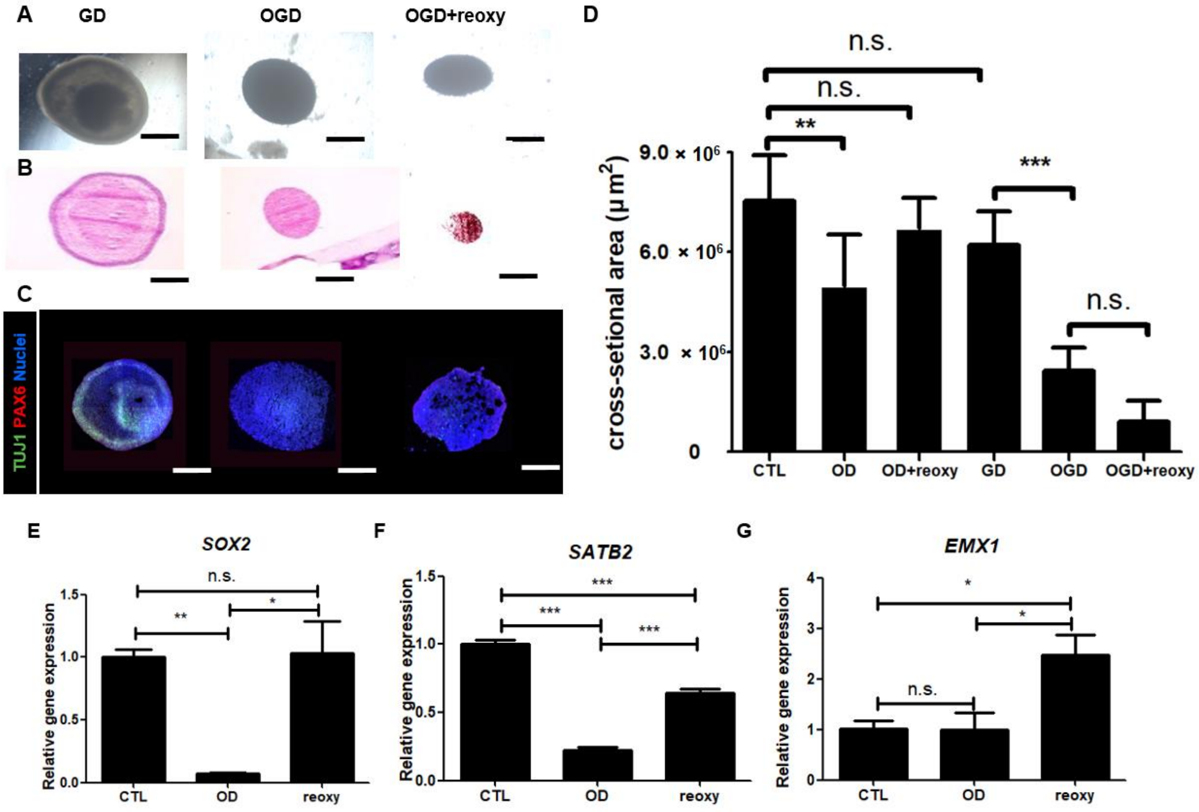

Supplementary Figure S2. Optimization of a brain hypoxic model using human 3D neural organoids (**A**) Representative organoid phase-contrast images of Glucose Deprivation (GD), Oxygen-Glucose Deprivation (OGD), and reoxygenation after Oxygen-Glucose Deprivation (OGD+reoxy). Scale bars, 1 mm (**B**) Hematoxylin and eosin staining image of GD, OGD, and OGD+repxy. Scale bars, 1 mm. (**C**) Confocal images show immature neuron marker (TUJ1) and radial glia cell marker (PAX6) in GD, OGD, and OGD+reoxy. Scale bars, 1mm. (**D**) Quantification of the cross-section area shows organoids of Control (CTL), oxygen deprivation (OD; red); reoxygenation after oxygen deprivation (OD+reoxy), Glucose Deprivation (GD), Oxygen-Glucose Deprivation (OGD), and reoxygenation after Oxygen-Glucose Deprivation (OGD+reoxy). Each group $n = 5$ organoids, mean ± SD, **$p < 0.01$, ***$p < 0.001$, n. s. not statistically significant versus Static. (**E–G**) Gene expression of radial glia cell *SOX2* (E); mature neuron *SATB2* (F); the developing cerebral cortex *EMX1* (G) in CTL, OD, and reoxy. Scale bars, 1mm.

Supplementary Table S1. List of antibodies

Antibody	Working Dilutions	Company	Catalog number
TBR1	1 : 500	Abcam	cat. # ab31940
TBR2	1 : 500	Abcam	cat. # ab23345
TBR2	1 : 500	Merck	cat. # AB15894
SOX2	1 : 500	Merck	cat. # AB5603
SATB2	1 : 500	Abcam	cat. # ab34735
Nestin	1 : 250	Abcam	cat. # ab22035
PAX6	1 : 250	Biolegend	cat. # PRB-278P
FOXG1	1 : 500	Abcam	cat. # ab18259
MAP2	1 : 500	Merck	cat. # MAB3418
CITP2	1 : 500	Abcam	cat. # ab18465
Reelin	1 : 500	Merck	cat. # MAB5366
OLIG2	1 : 500	Abcam	cat. # ab9610
O4	1 : 500	Millipore	cat. #MAB345
GFAP	1 : 500	Cell signaling	cat. # 3670S
Ki67	1 : 500	Abcam	cat. # ab15580
NeuN	1 : 500	Merck	cat. # MAB377
TUJ1	1:1000	Novus	cat. # NB100-1612
phospho-Vimentin	1:1000	MBL	cat. # D076-3S
Cleaved -Cas3	1 : 500	Cell signaling	cat. # 9661
Cleaved-PARP	1 : 500	Abcam	cat. # ab4830
Bcl-2	1 : 500	Santa Cruz Biotechnology	cat. # sc-7382
